# Profiling and Distribution of Metabolites of Procyanidin B2 in Mice by UPLC-DAD-ESI-IT-TOF-MS^n^ Technique

**DOI:** 10.3389/fphar.2017.00231

**Published:** 2017-05-04

**Authors:** Ying Xiao, Zhongzhi Hu, Zhiting Yin, Yiming Zhou, Taiyi Liu, Xiaoli Zhou, Dawei Chang

**Affiliations:** ^1^School of Perfume and Aroma Technology, Shanghai Institute of TechnologyShanghai, China; ^2^School of Food and Biological Engineering, Shaanxi University of Science and TechnologyXi'an, China

**Keywords:** procyanidin B2, metabolic profile, identification, distribution, UPLC-DAD-ESI -IT-TOF-MS^n^

## Abstract

The metabolite profiles and distributions of procyanidin B2 were qualitatively described using UPLC-DAD-ESI-IT-TOF-MS^n^ without help of reference standards, and a possible metabolic pathway was proposed in the present study. Summarily, 53 metabolites (24 new metabolites) were detected as metabolites of procyanidin B2, and 45 of them were tentatively identified. Twenty seven metabolites were assigned as similar metabolites of (−)-epicatechin by scission of the flavanol interflavanic bond C4–C8, including 16 aromatic metabolites, 5 conjugated metabolites, 3 ring-cleavage metabolites, and 2 phenylvalerolactone metabolites. Additionally, 14 metabolites were conjugates of free procyanidin B2, comprising 9 methylation metabolites, 8 sulfation metabolites, 5 hydration metabolites, 2 hydroxylation metabolites, 1 hydrogenation metabolites, and 1 glucuronidation metabolites. The results of metabolite distributions in organs indicated that the conjugated reaction of free procyanidin B2 mainly occurred in liver and diversified metabolites forms were observed in small intestine. The metabolic components of procyanidin B2 identified in mice provided useful information for further study of the bioactivity and mechanism of its action.

## Introduction

Proanthocyanidins are one subclass of polyphenolic compounds that can be detectable in a wide variety of natural sources, such as nuts, beans, apples, cocoa, and tea (Prior and Gu, 2005; Xu et al., 2010; Li et al., 2013). Proanthocyanidins have been known as one kind of strong antioxidant compounds to prevent or ameliorate metabolic syndrome and to reduce the incidence of cardiovascular diseases and cancer (Veluri et al., 2006; Terra et al., 2011; Guerrero et al., 2013; Margalef et al., 2015).

**Figure 1:**
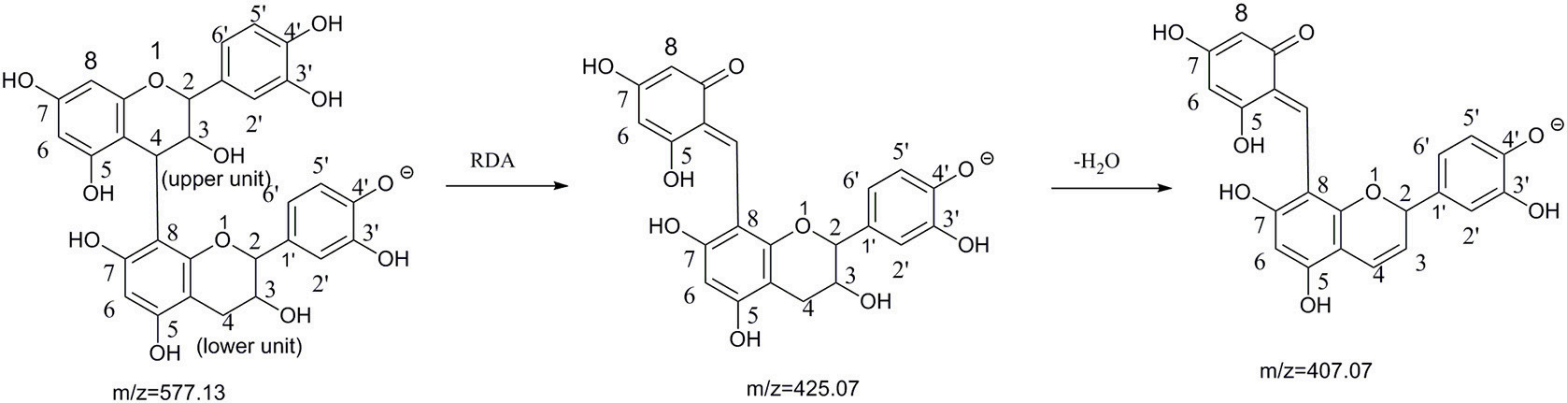
**The pathway of retro-Diels-Alder(RDA) cleavage of procyanidin B2 and binding site label (Sun and Miller, 2003; Stoupi et al., 2010b)**.

The most ubiquitous proanthocyanidin present in nature source is the procyanidin B2, where the flavan-3-ols are linked via an interflavan bond between the benzylic C-4 carbon of the heterocyclic ring of the upper unit and the C-8 carbon of the flavan-3-ol A-ring of the lower unit (4→8) (Figure 1; Stoupi et al., 2010a,c). There has been a great increase in knowledge about the bioactivities of procyanidins extraction from natural sources rich in procyanidin B2. However, the absorption and metabolism progress of procyanidin B2 was complex. Procyanidin B2 has been reported to be slightly absorbed without conjugation or methylation (Clifford, 2004; Appeldoorn et al., 2009b; Stoupi et al., 2010a). Interestingly, the beneficial effects of procyanidin B2 may in part be due to the metabolites formed in the tissue rather than the parent compounds (Del Rio et al., 2013; Guerrero et al., 2013). Furthermore, Spencer et al. (2001) found that (−)-epicatechin was the major metabolites of purified procyanidin B2 after transfer across the rat small intestine via LC-MS technology. Shoji et al. (2006) detected methylated B-type dimmer in rat plasma after oral administration apple procyanidins using the Porter method and LC-MS. However, the metabolites of procyanidin B2 have not been still completely clear, and only a few metabolites forms of procyanidin B2 were found *in vivo*, such as methylation, phenolic acids, and monomer forms.

To gain a more comprehensive understanding of the metabolism of procyanidin B2, it was necessary to profile metabolites and reveal the distribution of these metabolites *in vivo*, but the metabolites profiling of procyanidin B2 was difficult because of little structural information reported previously. Therefore, one powerful analysis technology is essential. In the past few years, triple quadrupole mass spectrometry coupled with chromatographic separation has become a powerful and frequently used technique for metabolite identification, whereas they are challenged for complex biological samples containing many compounds because of low sensitivity in full scan mode. Notably, limited reports using one novel strategy demonstrated that LC combined with hybrid ion trap/time-of flight mass spectrometry (LC-IT-TOF) could be effectively applied to discover the metabolites of polyphenolic compounds, such as (+)-catechin, (−)-epicatechin, and Paeoniae Radix Rubra extract (Liang et al., 2013, 2014). Undoubtedly, LC-IT-TOF technology characterized with a wide mass range, high sensitivity in full scan mode, high resolving power, and multistage fragmentations enables to the powerful identification of functional compounds undergoing multiple and unpredictable metabolites from biological matrix in lack of authentic compounds for verification (Ferrer and Thurman, 2007; Wang et al., 2009; Tian et al., 2013).

Therefore, the aims of this study were to explore more information of the *in vivo* metabolites of procyanidin B2 (Epicatechin-(4β → 8)-epicatechin), profile the metabolites in mice tissues, and analyze the possible metabolic pathways of procyanidin B2 via the UPLC-DAD-IT-TOF-MS^n^ technique without the help of metabolites reference standards.

## Materials and methods

### Chemicals and materials

Acetonitrile (HPLC-grade), methanol (HPLC-grade), and formic acid (HPLC-grade) were purchased from Aladdin Industrial Corporation. Ultrapure water was prepared by Milli-Q water purification system (Madrid, Spain). Sodium carboxymethyl cellulose (CMC-Na) was purchased from Aladdin Industrial Corporation. Standards of procyanidin B2 and (−)-epicatechin were purchased from Tauto Biotech Corporation. (Shanghai, China). The purities of all standards were over 99% based on HPLC analysis.

### Animal experiments

Eighteen male 10-week C57BL/6 mice (23 ± 2 g) were obtained from SLAC Laboratory Animal center (Shanghai, China). All animals were housed in an environmentally controlled breeding room (24 ± 2°C, 60 ± 10% humidity) for 7 days with unrestricted chow and water. Then, the mice were randomly divided into 2 groups with nine mice per group (Group A: the procyanidin B2 group and Group B: control group) before the beginning of experiments. The procyanidin B2 was dissolved in 0.5% CMC-Na water solution and orally administered to group A at a dose of 800 mg/kg body weight, and the mice in group B were administered 0.5% CMC-Na water solution in the same way. The mice were administered for 3 days at 9:00 am. All experiments were performed in compliance with the Chinese legislation on the use and care of laboratory animals and were approved by the Shanghai Institute of Technology Committee on Animal Care and Use.

### Sample collection and pretreatment

Urine sample was collected within 2 days after the first administration. Finally, all urine samples from one group were merged into one sample. Whereafter, the urine samples were dried by Boyikang FD-80 freezer dryer (Beijing, China). Next, 1.00 g of dried sample was redissolved by 2 mL methanol. Then, the solution was centrifuged at 2,795 g for 15 min (Sigma 3k-18, Sigma, Germany) after ultrasonic bath for 30 min. Afterward, the supernatant was stored in −80°C(MDF-1156, SANYO, Japan) before the next step of analysis (Liang et al., 2013).

The blood samples were collected in heparinized tubes at 1, 1.5, and 2 h by extracting eye bowl after the first administration of the last day under anesthesia state, and then centrifuged for 15 min at 1,789 g for obtaining the plasma samples. All plasma samples from one group were merged into one sample. Finally, each plasma sample (3 ml) was deproteinized with ice-cold acetonitrile (9 ml) in an ultrasonic bath for 20 min, and then was centrifuged at 2,795 g for 15 min to remove precipitated protein, and the solid residue was washed for three times using ice-cold acetonitrile (1 ml). Then, the merged supernatant was dried with a gentle flow of nitrogen in ice bath. The residue was redissolved in 3 ml methanol and was centrifuged at 2,795 g for 15 min after vortex oscillation. The supernatant was reconstituted to dryness, and was quickly redissolved in 200 μL methanol with vortex oscillation. The treated samples were centrifuged at 11,180 g for 10 min before injecting the system of high-performance liquid chromatography.

The pretreatment of heart, liver, brain, spleen, lungs, kidneys, or small intestine was homogenized by a homogenizer (PRO 200, Oxford, USA), which was suspended in methanol (1/10, g/ml). Then, the homogenates were in the ice-cold ultrasonic bath for 30 min and centrifuged at 2,795 g for 15 min. Afterward, the supernatant was evaporated to dryness in vacuum at 45°C, and then quickly redissolved in 2 mL methanol. The pretreated samples were stored at −80°C before analysis. All the samples were filtered through 0.45 μm nylon membrane before injecting the system (Liang et al., 2013).

### Instruments and conditions

The UPLC-DAD-ESI-IT-TOF analysis of the metabolites was performed using Shimadzu LC-30AD instrument equipped with two LC-30AD pumps, an SIL-30AC autosampler, SPD-M20A, CTO-20AC column oven, and CBM-20A system controller, and coupled to an IT-TOF mass spectrometer with an ESI interface.

The chromatography separations were performed on a Shim-pack HR-ODS column (150 mm × 2.1 mm, 3 μm). The column was eluted with a gradient mobile phase A of water-formic acid (100: 0.1, v/v) and B of acetonitrile, The elution gradient was 0–2 min, 5–10% B, 2–4 min, 10–12% B, 4–7 min, 12–18% B, 7–13 min, 18–27% B, 13–32 min, 27–56% B, 32–37 min, 56–100% B, and 37–47 min 100% B. The flow rate was 0.2 mL/min; the injection volume was 5 μL and the column oven temperature was kept at 30°C.

The tandem mass spectrometry analyses were carried out on an IT-TOF (Shimadzu, Japan) with the full scan over m/z 100–900 (MS^1^) and m/z 50–900 (MS^2^ and MS^3^) in the ion model of negative(NI) and positive(PI). The parameters were as follows: heat block and curved desolvation line temperature, 200°C; nebulizing nitrogen gas flow, 1.5 L/min; interface voltage: (+), 4.5 kv; (−), 3.5 kv; detector voltage, 1.56 kv; relative collision-induced dissociation energy, 50%.

### Methods of analyzing the metabolites

To find more information of metabolites, in this study, we adopted a specially pattern for metabolites screening and identification. The procedures included three steps, (1) collecting the information of metabolites of procyanidin B2 by searching the literature databases and bioinformatic databases on line (Chemspider: http://www.chemspider.com, http://metlin.scripps.edu/); (2) searching the possible mass-to-charge ratio of metabolites by extracted ion chromatograms (EICs) in the total ion chromatograms (TICs) comparing with the TICs of control group using Profiling Solution version 3.60 and metabolites with the aid of MetlID Solution 1.2 software; (3) the known metabolites were confirmed by the databases described above. The unknown metabolites were tentatively identified in several steps: firstly, the type of substitution was identified based on the characteristic fragment ions and neutral losses in MS^n^ spectrum; second, the skeleton structure was speculated in comparison with the mass fragmentation patterns of reference standard procyanidin B2; third, the proposed isomeric metabolites were further confirmed by characteristic fragment ions in MS^n^ spectrum, n-octanol/water partition coefficient (ClogP value) and predominant conjugation site reported by other literature (Liang et al., 2013, 2014). All the data was processed by Shimadzu software of Formula Predictor version 1.20.

## Results and discussion

### Fragmentation behaviors of the reference compounds in negative ion mode

**Figure 2:**
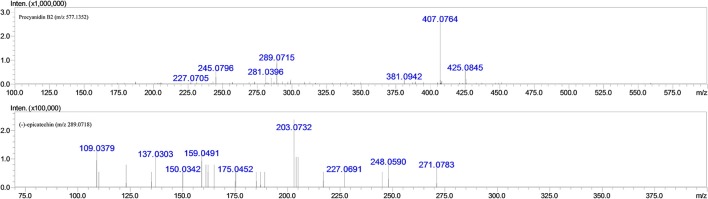
**MS^**2**^ fragmentation data obtained in negative ion detection mode of procyanidin B2 and (−)-epicatechin**.

Since it has been well proven that the mass fragmentation patterns of metabolites are always similar to the parent compounds, the fragmentation patterns analysis of parent compound is very helpful for the metabolite characterizations. Therefore, this study conducted a detailed fragmentation of procyanidin B2 and (−)-epicatechin authentic standard in UPLC-DAD-ESI-IT-TOF (shown in Figure 2), which provided guidance in the subsequent structure of metabolites. Procyanidin B2 showed the [M-H]^−^ at m/z 577.1260 (C_30_H_25_O_12_), which was broken into characteristic fragment ions at m/z 425.0845 (C_22_H_17_O_9_), 407.0764 (C_22_H_15_O_8_), 289.0715 (C_15_H_14_O_6_), and m/z 245.0796 (C_14_H_14_O_4_) in the MS^2^ spectrum. The (−)-epicatechin showed the characteristic fragment ions at m/z 245.0799 (C_14_H_14_O_4_), 205.0476 (C_11_H_10_O_4_), 137.0258 (C_7_H_6_O_3_), and m/z 125.0258 (C_6_H_6_O_3_).

**Figure 3:**
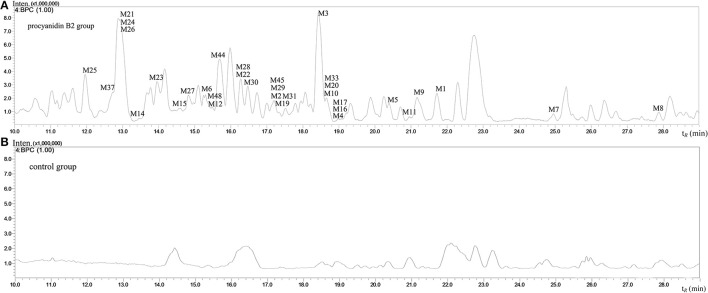
**The negative ion base peak chromatograms of mice urine. (A)** The BPC of urine of Procyanidin B2 group; **(B)** The BPC of urine of control group.

**Figure 4:**
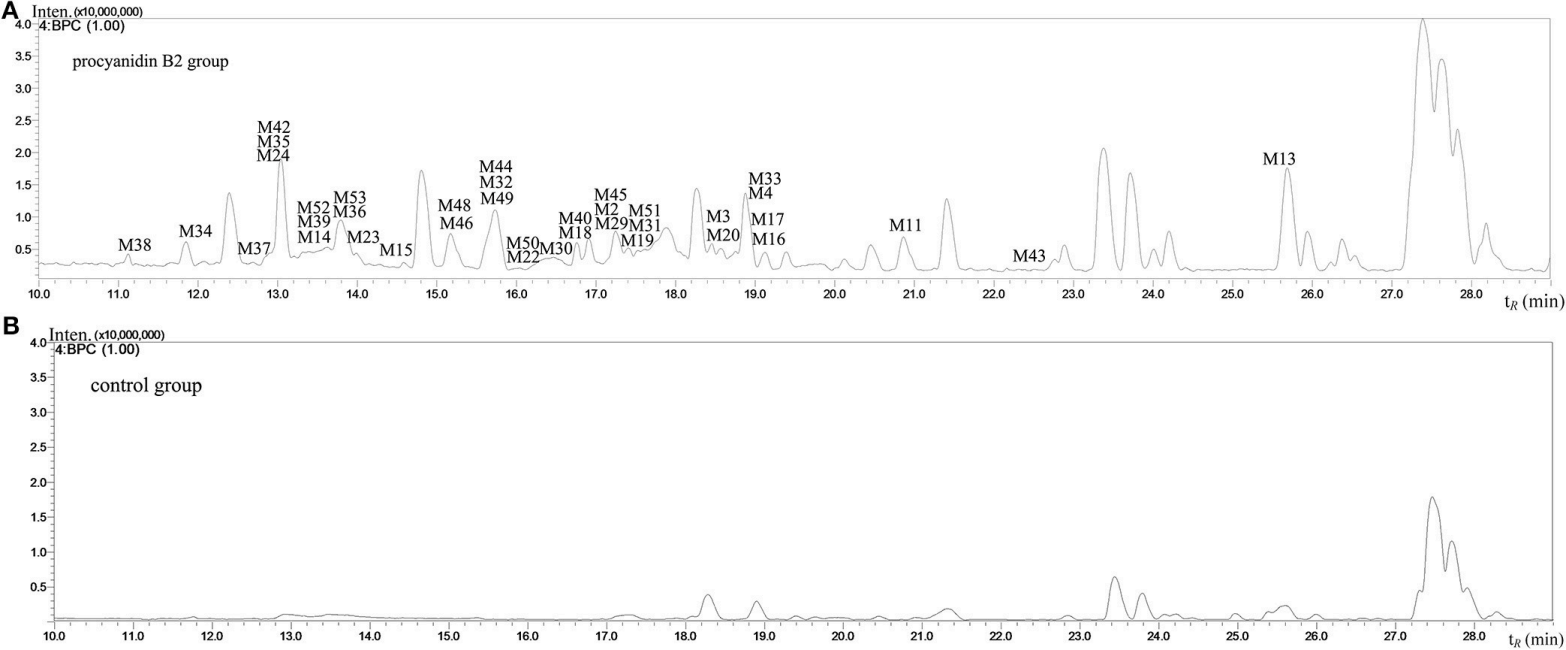
**The negative ion base peak chromatograms of mice plasma. (A)** The BPC of plasma of Procyanidin B2 group; **(B)** The BPC of plasma of control group.

The MS full-scan might reveal all the ion chromatograms of the expected or unexpected metabolites. The peaks detected in the ion chromatograms of sample group correspond to the mass-to-charge ratio (m/z) of the possible metabolites compared with the ion chromatograms of control group (shown in Figures 3 and 4). According to predicted gains and neutral losses in molecular masses of the metabolites compared with the molecular mass of the parent compound, the multiple metabolites from biological matrix could be effectively revealed based on the advantage of LC-IT-TOF technology in absence of authentic standards for calibration compounds.

### Characterization of metabolites of procyanidin B2

A total of 53 compounds were detected as metabolites of procyanidin B2 (45 of them were identified). As for 45 preliminarily identified metabolites, 27 metabolites (M_r_ < 577.13) were degraded from procyanidin B2 by cleavage of the flavanol interflavanic bond C4–C8. Additionally, 14 conjugates were formed, including 9 methylation metabolites, 8 sulfation conjugates, 5 hydration metabolites, 2 hydroxylation metabolites, 1 hydrogenation metabolite, and 1 glucuronidation metabolite. Interestingly, 24 compounds as metabolites of procyanidin B2 were newly found, including 8 methylation metabolites, 9 sulfation conjugates, 2 hydroxylation metabolites, 7 hydration metabolites, 1 hydrogenation metabolite, 1 glucuronidation metabolite, and 8 unidentified metabolites. The data was summarized in Tables 1, 2.

**Table 1:** **MS data obtained in negative ion detection mode of 45 identification metabolites in urine, plasma, and organ distributions of mice**.

**No**.	**t_R_ (min)**	**Formula**	**Measured (Da)**	**Predicted (Da)**	**Error (ppm)**	**DBE**	**U**	**P**	**I**	**L**	**H**	**S**	**LU**	**K**	**B**	**Identification**
M1	21.9	C_15_H_14_O_6_	289.0728	289.0718	3.46	9	+	−	+	−	−	−	−	−	−	(−)-Epicatechin
M2	17.2	C_15_H_14_O_9_S	369.0307	369.0286	5.69	9	+	+	−	−	+	+	+	+	−	(−)-Epicatechin-5/7-O-sulfate
M3	18.5	C_15_H_14_O_9_S	369.0307	369.0286	5.69	9	+	+	−	−	+	+	+	−	−	(−)-Epicatechin-5/7-O-sulfate
M4	19.0	C_16_H_16_O_9_S	383.0423	383.0442	−4.96	9	+	+	+	−	+	−	−	+	−	3′-O-Methyl-(−)-Epicatechin-5/7-O-sulfate
M5	20.5	C_16_H_16_O_9_S	383.0429	383.0442	−3.39	9	+	−	+	−	−	−	−	−	−	3′-O-Methyl-(−)-Epicatechin-5/7-O-sulfate
M6	15.3	C_21_H_22_O_15_S	545.0593	545.0607	−2.75	11	+	−	−	−	−	−	−	−	−	5/7-O-Sulfate-(−)-Epicatechin-glucuronide
M7	25.0	C_15_H_16_O_6_	291.0870	291.0874	−1.37	8	+	−	−	−	−	−	−	−	−	3,4-diHPP-2-ol
M8	27.7	C_21_H_24_O_12_	467.1209	467.1195	3.00	10	+	−	−	−	−	−	−	−	−	5-O-Glucuronide-3,4-diHPP-2-ol
M9^*^	21.2	C_15_H_18_O_7_	309.0964	309.0980	−5.18	7	+	−	−	−	−	−	−	−	−	3,4-diHPP-2-ol(·2H_2_O)
M10	18.6	C_11_H_12_O_7_S	287.0237	287.0231	2.09	6	+	−	+	−	−	−	−	−	−	5-(3,4-Dihydroxypheny)-γ-valerolactone sulfate
M11	20.8	C_11_H_12_O_7_S	287.0229	287.0231	−0.70	6	+	+	+	−	−	−	−	−	−	5-(3,4-Dihydroxypheny)-γ-valerolactone sulfate
M12	15.5	C_11_H_14_O_8_S	305.0346	305.0337	2.95	5	+	−	−	−	−	−	−	−	−	5-(3,4-Dihydroxypheny)-4-hydroxypentanoic sulfate
M13	25.5	C_12_H_16_O_8_S	319.0476	319.0493	−5.33	5	−	+	−	−	−	−	−	−	−	3'-O-Methyl-5-(3,4-dihydroxyphenyl)-4-hydroxypentanoic sulfate
M14	13.5	C_9_H_10_O_7_S	261.0055	261.0074	−7.28	5	+	+	−	−	−	−	−	−	−	3,4-Dihydroxyphenylpropionic acid sulfate
M15	14.6	C_9_H_10_O_7_S	261.0063	261.0074	−4.21	5	+	+	−	−	−	−	−	−	−	3,4-Dihydroxyphenylpropionic acid sulfate
M16	19.1	C_10_H_10_O_7_S	273.0066	273.0074	−2.93	6	+	+	+	−	+	−	−	−	−	Ferulic acid sulfate
M17	19.0	C_9_H_10_O_6_S	245.0136	245.0125	4.49	5	+	+	−	−	−	−	−	−	−	3-Hydroxy phenylpropionic acid sulfate
M18	16.7	C_9_H_10_O_6_S	245.0135	245.0125	4.08	5	−	+	−	−	−	−	−	−	−	4-Hydroxy phenylpropionic acid sulfate
M19	17.3	C_9_H_8_O_6_S	242.9971	242.9969	0.82	6	+	+	+	−	+	−	−	−	−	M-Coumaric sulfate
M20	18.6	C_9_H_8_O_6_S	242.9966	242.9969	−1.23	6	+	+	−	−	+	−	−	−	−	P-Coumaric sulfate
M21	13.1	C_8_H_8_O_7_S	246.9910	246.9918	−3.24	5	+	−	−	−	+	−	−	−	−	3,4-Dihydroxyphenylacetic acid sulfate
M22	16.2	C_8_H_8_O_7_S	246.9909	246.9918	−3.64	5	+	+	+	−	−	−	−	−	−	3,4-Dihydroxyphenylacetic acid sulfate
M23	14.0	C_8_H_8_O_6_S	230.9981	230.9969	5.19	5	+	+	+	−	−	−	−	−	−	3-Hydroxyphenylacetic acid sulfate
M24	13.0	C_7_H_6_O_7_S	232.9775	232.9761	6.01	5	+	+	−	−	−	−	−	−	−	3-O-Protocatechuic acid sulfate
M25	12.0	C_7_H_6_O_7_S	232.9777	232.9761	6.87	5	+	−	−	−	−	−	−	−	−	3-O-Protocatechuic acid sulfate
M26	13.0	C_7_H_6_O_6_S	216.9814	216.9812	0.92	5	+	−	−	−	−	−	−	−	−	3-Hydroxybenzoic acid sulfate
M27	14.7	C_7_H_6_O_6_S	216.9812	216.9812	0	5	+	−	−	−	−	−	−	−	−	4-Hydroxybenzoic acid sulfate
M28^*^	16.2	C_30_H_27_O_12_	578.1465	578.1430	8.05	17.5	+	−	−	−	−	−	−	−	−	Hydrogenation procyanidin B2
M29	17.2	C_31_H_28_O_12_	591.1513	591.1508	0.85	18	+	+	+	+	−	−	−	−	−	3'-O-Methyl upper procyanidin B2
M30	16.5	C_31_H_28_O_12_	591.1513	591.1508	0.85	18	+	+	+	+	−	−	−	+	−	4'-O-Methyl upper procyanidin B2
M31	17.4	C_31_H_28_O_12_	591.1513	591.1508	0.85	18	+	+	+	−	−	−	−	−	−	3′/4′-O-Methyl lower procyanidin B2
M32^*^	15.8	C_32_H_30_O_12_	605.1659	605.1665	−0.99	18	−	+	+	+	+	−	−	+	−	3'-O-Methyl upper-3′/4′-methyl lower procyanidin B2
M33^*^	18.7	C_32_H_34_O_14_	641.1801	641.1876	−11.7	16	+	+	−	+	−	−	−	−	−	3'-O-Methyl upper-3′/4′-methyl lower procyanidin B2(·2H_2_O)
M34^*^	11.7	C_30_H_26_O_15_S	657.0901	657.0920	−2.89	18	−	+	+	−	−	−	−	−	−	Procyanidin B2 sulfate
M35^*^	12.9	C_30_H_26_O_15_S	657.0901	657.0920	−2.89	18	−	+	+	−	−	−	−	−	−	Procyanidin B2 sulfate
M36^*^	13.7	C_30_H_26_O_15_S	657.0901	657.0920	−2.89	18	−	+	+	−	−	−	−	−	−	Procyanidin B2 sulfate
M37^*^	12.7	C_38_H_38_O_18_	781.2002	781.2003	−0.13	18	+	+	−	−	−	−	−	−	−	3′-O-Methyl upper-3′/4′-methyl lower procyanidin B2 glucuronide
M38^*^	11.1	C_31_H_30_O_16_S	689.1220	689.1182	6.97	17	−	+	−	+	+	−	−	−	−	3′/4′-O-Methyl lower procyanidin B2(·H_2_O) sulfate
M39^*^	13.4	C_31_H_30_O_16_S	689.1230	689.1182	4.79	17	−	+	−	+	+	−	−	−	−	3′/4′-O-Methyl lower procyanidin B2(·H_2_O) sulfate
M40^*^	16.8	C_31_H_30_O_16_S	689.1215	689.1182	5.83	17	−	+	−	+	−	−	−	−	−	3′/4′-O-Methyl lower procyanidin B2(·H_2_O) sulfate
M41^*^	16.8	C_32_H_32_O_16_S	703.1379	703.1338	5.83	17	−	+	−	+	−	−	−	−	−	3′-O-Methyl upper-3′/4′-methyl-lower procyanidin B2(·H_2_O) sulfate
M42^*^	12.8	C_30_H_26_O_16_S	673.0869	673.0869	0	18	−	+	−	−	−	−	−	−	−	6/8-Hydroxy upper procyanidin B2 sulfate
M43^*^	22.5	C_30_H_26_O_16_S	673.0869	673.0869	0	18	−	+	−	−	−	−	−	−	−	6/8-Hydroxy upper procyanidin B2 sulfate
M44^*^	15.6	C_30_H_30_O_14_	613.1550	613.1563	−2.12	16	+	+	+	+	+	+	+	+	+	Procyanidin B2(·2H_2_O)
M45^*^	17.2	C_31_H_30_O_12_	593.1660	593.1665	−0.84	17	+	+	+	−	−	−	−	−	−	Methyl C-ring cleavage procyanidin B2

* *New metabolites of procyanidin B2.DBE, Double bond equivalent; U, urine; P, plasma; H, heart; L, liver; S, spleen; LU, lung; K, kidney; B, brain; I, small intestine; +, detected; −, undetected*.

**Table 2:** **MS^**n**^ data obtained in negative ion detection mode of 8 unidentified metabolites in urine, plasma, and organ distributions of mice**.

**No**.	**t_*R*_ (min)**	**Measured (Da)**	**U**	**P**	**I**	**L**	**H**	**S**	**LU**	**K**	**B**	**Major fragment ions detected in NI mode**
M46^*^	9.5	702.1426	−	−	+	−	−	−	−	−	−	684.2828, 577.1286, 451.1019, 289.0721
M47^*^	15.1	676.1156	−	+	+	−	−	−	−	−	−	577.1277, 451.1068, 425.0806, 407.0690, 245.0768
M48^*^	15.5	691.1194	+	+	+	+	−	−	−	+	−	612.1849, 577.1322, 451.1019, 419.0753, 289.0707, 301.0761
M49^*^	16.8	685.1241	−	−	+	−	−	−	−	−	−	603.1468, 527.3209, 493.1042, 451.0964, 289.0707
M50^*^	16.0	705.1387	−	+	−	+	−	−	−	−	−	591.1429, 425.0825, 407.0690, 301.0675, 289.0660, 247.0580
M51^*^	17.6	627.1335	−	+	−	+	−	−	−	−	−	591.1424, 301,0760, 289.0769
M52^*^	13.5	714.1452	−	+	−	+	+	−	−	+	−	589.1334, 451.1008, 437.0857, 419.0753, 289.0707, 237.0593
M53^*^	13.6	325.0501	−	+	+	−	−	−	−	−	−	306.1210, 289.0721, 245.0897, 205.0464, 165.0126

* *New metabolites of procyanidin B2.DBE, Double bond equivalent; U, urine; P, plasma; H, heart; L, liver; S, spleen; LU, lung; K, kidney; B, brain; I, small intestine; +, detected; −, undetected*.

### Identification of the monomer metabolites: M1

M1 was calculated to be C_15_H_14_O_6_ on the basis of high-resolution mass spectrometry (HRMS) data. Since the typical fragment ions at m/z 245.07, 205.04, and 137.02 were noticed, which were in high accordance with the reference (−)-epicatechin, as generated by Retro-Diels-Alder (RDA) cleavage in accordingly with the previous reports (Liang et al., 2014), the M1 was confirmed as (−)-epicatechin formed by cleavage of interflavanic bond of procyanidin B2.

### Conjugated metabolites of (−)-epicatechin: M2–M6

In the MS^n^ spectra of M2-M6, the same fragmentation ions at m/z 289.06, 245.07, and 137.02 were found, which were similar with the fragment behaviors of (−)**-**epicatechin. Therefore, M2-M6 were the metabolic compounds of (−)**-**epicatechin. The molecular formula of M2 and M3 showed the same [M-H]^−^ ion at m/z 369.03, which could fragment into [M-SO_3_-H]^−^ at m/z 289.07 by neutral loss of 79.95 Da. Since the typical A-ring fragment ions at m/z 216.98 and 137.02 of M2 and M3 were noticed, which were produced by RDA cleavage, we could deduce that the sulfonic group should be conjugated at A-ring. Hence, it was concluded that M2 and M3 were (−)-epicatechin-5/7-O-sulfate. M4 and M5 manifested the same [M-H]^−^ at m/z 383.04 in their MS^n^ spectra, and the same [M-CH_2_-H]^−^ at m/z 369.03 was detected by neutral loss of 14.01 Da (elemental composition: CH_2_). Accordingly, M4 and M5 were presumed to be methylation M2/M3 with the preferable site of C-3′-OH of 3′-O-methyl-5/7-O-sulfate (−)-epicatechin (Selma et al., 2009; Liang et al., 2014). Because the molecular formula of M6 was determined to be C_21_H_22_O_15_S with the [M-C_6_H_8_O_6_-H]^−^ at m/z 369.03 by a neutral loss of 176.03 Da (elemental composition: C_6_H_8_O_6_) in the NI MS^2^ spectra, M6 was supposed to be one metabolite of (−)**-**epicatechin, which was formed through glucuronidation and sulfation. But we could not determine its exact binding site, so it was tentatively inferred to be 5/7-O-sulfate-epicatechin-O-glucuronide (Liang et al., 2014).

### Metabolites from C-ring cleavage of (−)-epicatechin monomer: M7–M9

The molecular formulas of M7-M9 were calculated to be C_15_H_16_O_6_, C_21_H_24_O_12_, and C_15_H_18_O_7_, respectively, with the [M-H]^−^ at m/z 291.08, 467.12, and 309.09 based on the HRMS data. In the NI MS^n^ spectra, M7, M8, and M9 had the same characteristic fragment ions at m/z 247.09 (elemental composition: C_14_H_15_O_4_), 167.03 (elemental composition: C_8_H_7_O_4_), and 123.04 (elemental composition: C_7_H_7_O_2_), showing the similar fragmentation pathways of 1-(3′,4′-dihydroxyphenyl)-3-(2′,4′, 6′-trihydroxyphenyl)-propan-2-ol (3, 4-diHPP-2-ol) (Liang et al., 2014; Margalef et al., 2014). As a result, M7 was assigned as 3,4-diHPP-2-ol. M8 showed the [M-C_6_H_8_O_6_-H]^−^ at m/z 291.08 by neutral loss 176.03 Da (elemental composition: C_6_H_8_O_6_), so it was preliminarily identified as glucuronic acid conjugates of 3,4-diHPP-2-ol. According to the glucuronidation of (−)**-**epicatechin preferably occurred at the C-5-OH of A-ring (Shali et al., 1991), M8 was tentatively identified as 5-O-glucuronide-3,4-diHPP-2-ol. As for M9, the neutral loss of 18.01 Da (elemental composition: H_2_O) was observed. Therefore, M9 was identified as hydration products of 3,4-diHPP-2-ol.

### Phenylvalerolactone metabolites: M10–M11

The molecular formula of M10-M11 were calculated to be C_11_H_12_O_7_S with the same [M-SO_3_-H]^−^ at m/z 207.06 by the neutral loss of 79.95 Da (elemental composition: SO_3_), and the precursor ion [M-SO_3_-H]^−^ at m/z 207.06 can break into characteristic fragment ions at m/z 163.07, and m/z 122.04. Moreover, the fragmentation pathways of the aglucon were in good accordance with that of 5-(3,4-dihydroxyphenyl)-γ-valerolactone (Appeldoorn et al., 2009a; Urpi-Sarda et al., 2009). Thus, they were confirmed to be 5-(3,4-dihydroxyphenyl)-γ-valerolactone sulfate.

### Aromatic acid metabolites: M12–M27

**Figure 5:**
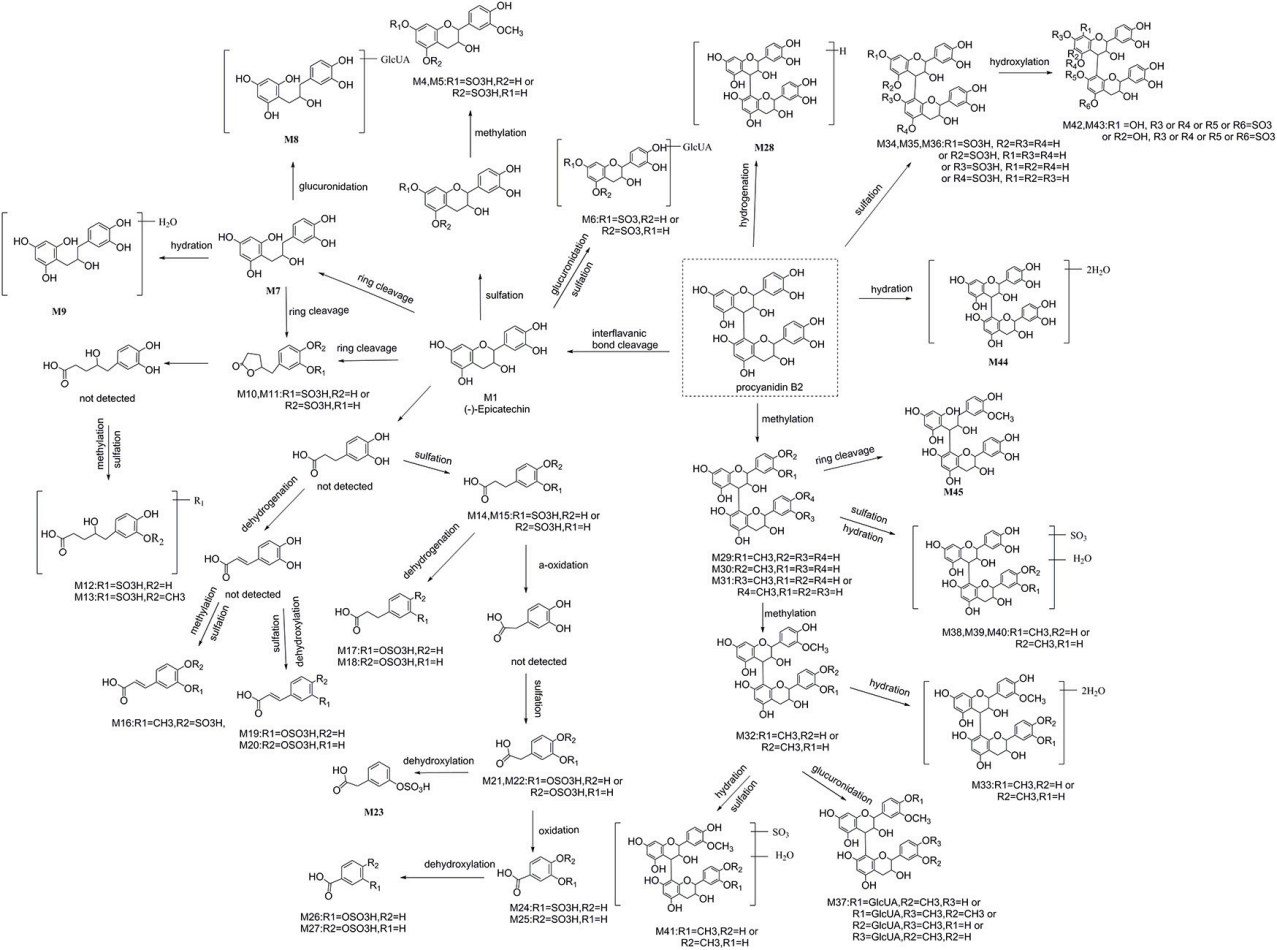
**The proposed metabolic pathway of 45 identified metabolites of procyanidin B2 in mice**.

Summarily, 16 aromatic acid metabolites are tentatively identified, including 2 phenylvalenic acid metabolites, 7 phenylpropionic acid metabolites, 3 phenylacetic acids metabolites, and 4 benzoic acid metabolites. Their structures are shown in Figure 5.

#### Phenylvalenic acid metabolites: M12–M13

The molecular formula of M12 was C_11_H_14_O_8_S with [M-H]^−^ at m/z 305.03, yielding fragmentation ions at m/z 225.07, 207.06, and 163.07, which were same with the characteristic fragment ions of 5-(3,4-dihydroxyphenyl)-γ-valerolactone. Since the neutral loss of 79.95 Da (elemental composition: SO_3_) was observed in their HRMS spectra, M12 was tentatively identified as the ring open product of 5-(3,4-dihydroxyphenyl)-γ-valerolactone sulfate with molecular formula of C_11_H_12_O_7_S of 5-(3,4-dihydroxyphenyl)-4-hydroxypentanoic acid sulfate (Liang et al., 2013). As the molecular weight of M13 was 14.01 Da (elemental composition: CH_2_) larger than M12, M13 was determined to be methyl M12, Furthermore, according to the major methylation site of (−)-epicatechin confirmed by the previous reports, the methyl conjugates site was on the C-3 position of M12 with the similarly conjugation rule (De Pascual-Teresa et al., 2000; Ou and Gu, 2014).

#### Phenylpropionic acid metabolites: M14–M20

In the NI MS^n^ spectra of M14 and M15, [M-SO_3_-H]^−^ at m/z 181.05 formed by the neutral loss of 79.95Da (elemental composition: SO_3_) was detected, the characteristic ions yielded by aglucon at m/z 137.06, and 122.04 in the MS^n^ spectra were similar with the fragment behaviors of the 2-(3-hydroxy-4-methoxyphenyl) acetic acid (Liang et al., 2014). Hence, M14 and M15 were provisionally identified as 3,4-dihyroxyphenylpropionic acid sulfate. M16 showed molecular ion [M-H]^−^ at m/z 273.00, indicating that it was calculated to be C_10_H_10_O_7_S. Furthermore, the characteristic fragmentation ions at m/z 193.05 and 134.04 were observed, which were in high accordance with those of the ferulic acid according to the previous literature (Liang et al., 2014). Accordingly, M16 was identified to be ferulic acid sulfate. The molecular formula of M17 and M18 were determined to be C_9_H_10_O_6_S based on their HRMS data. In their NI MS^n^ spectra, the [M-SO_3_-H]^−^ at m/z 165.05 was detected, implying the neutral loss of 79.95 Da (elemental composition: SO_3_). Moreover, the fragment of [M-SO_3_-H]^−^ was identical to be hydroxy phenylpropionic acid because of previously reported (Urpi-Sarda et al., 2009). Therefore, M17 and M18 were identified as hydroxy phenylpropionic acid sulfate. Furthermore, Gonthier et al. reported that 3-hydroxy phenylpropionic acid was a preferred metabolite of (−)-epicatechin (Gonthier et al., 2003). Meanwhile, the peak area of M17 (peak area: 381, 733) was much larger than M18 (peak area: 268, 056). Accordingly, we assigned M17 as 3-hydroxy phenylpropionic acid sulfate and M18 as 4-hydroxyphenylpropionic acid sulfate. The molecular formula of M19 and M20 were identified as C_9_H_8_O_6_S based on the HRMS data. In their NI MS^n^ spectra, the [M-SO_3_-H]^−^ at m/z 163.04 was observed, which was formed by a neutral loss of 79.95 Da (elemental composition: SO_3_). The fragmentation behaviors of [M-SO_3_-H]^−^ were the same as coumaric acid. Moreover, the m-coumaric acid was preferred metabolite in mice in comparison with p-coumaric acid (Touriño et al., 2011). Hence, M19 with a bigger peak area (peak area: 110, 777) was found to be m-coumaric sulfate, and the M20 (peak area: 69, 181) was identified as p-coumaric sulfate.

#### Phenylacetic acids metabolites: M21–M23

M21 and M22 showed the same [M-H]^−^ ion at m/z 246.99, and yielded fragment ions at m/z 167.03 formed by the neutral loss of 79.95 Da (elemental composition: SO_3_). In addition, the characteristic fragmentation ions at m/z 123.04 of 3,4-dihydroxyphenylacetic acid for only characteristic catabolism of procyanidin B2 via á-oxidation of 3,4-dihydroxyphenylpropionic acid was also observed (Spencer et al., 2000; Gu et al., 2004). Hence, M21 and M22 were 3,4-dihydroxyphenylacetic acid sulfate. The molecular ion [M-H]^−^ of M23 at m/z 230.99 was observed in the MS^n^ spectra, which yielded the fragment ions at m/z 151.04, generated by a neutral loss of 79.95 Da (elemental composition: SO_3_). Moreover, the characteristic fragmentation at m/z 107.05 of 3-hydroxyphenylacetic acid was also detected in the MS^n^ spectra of M23 (Urpi-Sarda et al., 2009). As results, M23 was determined to be 3-hydroxyphenylacetic acid sulfate.

#### Benzoic acid metabolites: M24–M27

M24 and M25 showed the same molecular ion at m/z 232.97, and yielded [M-SO_3_-H]^−^ at m/z 153.02 in their NI MS^n^ spectra, which was formed by a neutral loss of 79.95 Da (elemental composition: SO_3_), suggesting that they were sulfate conjugates. Moreover, the [M-SO_3_-H]^−^ was identified to be protocatechuic acid comparing with the previous studies. Therefore, M24 (peak area: 1, 692, 371) was identified as 3-O-protocatechuic acid sulfate, while M25 (peak area: 624, 480) was assigned as 4-O-protocatechuic acid sulfate according to the previous reports (Vasudevan and Mahadevan, 1990; Urpi-Sarda et al., 2009). M26 and M27 showed the same molecular ion [M-H]^−^ at m/z 216.98 with the molecular formula of C_7_H_6_O_6_S, and yielded fragment ions at m/z 137.02 and m/z 93.03, which were the characteristic fragment ions of 3/4-hydroxybenzoic acid (Gonthier et al., 2003). Accordingly, they were identified as 3/4-hydroxybenzoic acid sulfate. Moreover, 3-hydroxybenzoic acid sulfate had a smaller ClogP (−1.409) than 4-hydroxybenzoic acid sulfate (ClogP = 0.345), which suggested that the former should have a shorter retention time. Accordingly, M26 (t_R_ = 13.0 min) was tentatively identified as 3-hydroxybenzoic acid sulfate, and M27 (t_R_ = 17.7 min) was 4-hydroxybenzoic acid sulfate.

### Conjugated metabolites of procyanidin B2 (Mr > 577): M28–M44

A total of 17 conjugated metabolites were identified, which had the same characteristic fragmentation behaviors at m/z 425.08 and 407.07, as generated from RDA cleavage (Shown in Figure 1) of procyanidin B2.

#### Hydrogenation reaction of procyanidin B2: M28

M28 showed the molecular ion at 578.14, and yielded the [M-2H]^−^ at m/z 577.13 (elemental composition: C_30_H_26_O_12_), which could produce characteristic fragmentations of m/z 425.08, 407.07, and 289.07. And the fragmentation behaviors of the aglucon were identical to that of procyanidin B2 by comparison with the reference compounds. Moreover, M28 increased the metabolite mass by 1.01 Da in the structure of procyanidin B2. As a result, M28 was inferred to be product of procyanidin B2 hydrogenation reaction.

#### Metabolites formed through methylation: M29–M33

M29, M30, and M31 had the same molecular ion at m/z 591.15, and the [M-CH_2_-H]^−^ at m/z 577.13 was observed by a neutral loss of 14.01 (elemental composition: CH_2_) in their NI MS^n^ spectra. In the NI MS^n^ spectra of M29 and M30, the fragment ions of m/z 303.08, 407.07, and 289.06 were also detected. Therefore, the methylation site was likely on the B-ring of upper (−)**-**epicatechin unit of procyanidin B2. According to the previously confirmed major methylation site of (−)**-**epicatechin of 3′-OH, similarly, M29 (peak area: 252, 351) was tentatively identified as 3′-O-methyl upper (−)**-**epicatechin unit of procyanidin B2 because of its massive quantity, and M30 (peak area: 172, 287) was determined to be 4′-O-methyl upper (−)**-**epicatechin unit (Shali et al., 1991; Stoupi et al., 2010a). Based on the MS^n^ data of M31, the characteristic fragment ions at m/z 407.07 and 421.04 were observed, which were formed by RDA cleavage (shown in Figure 1). Accordingly, M31 was tentatively identified to be 3′/4′-O-methyl lower (−)**-**epicatechin unit. Based on the HRMS data of M32, the molecular formula of the metabolites was determined to be C_32_H_30_O_12_, with two additional CH_2_ units in comparison with procyanidin B2. In the NI MS^n^ spectra of M32, the characteristic fragment ions at m/z 407.08 and 421.09 were also observed. Hence, it could be concluded that a CH_2_ unit should be in the 3′-OH or 4′-OH of lower (−)**-**epicatechin unit. According to the confirmed major methylation site of M29, M32 was temporarily identified as 3′-O-methyl upper (−)**-**epicatechin-3′/4′-methyl lower (−)**-**epicatechin.

The molecular formula of M33 was C_32_H_34_O_14_, as calculated from its HRMS data with m/z at 641.18, which yielded [M-2H_2_O-H]^−^ at m/z 605.16 formed by a neutral loss of 36.02 Da (elemental composition: H_4_O_2_) in the NI MS^n^ spectrum. Therefore, M33 was tentatively identified as double hydration M32.

#### Metabolites formed through sulfation: M34–M36

In the NI MS^n^ spectra of M34, M35, and M36, the same [M-SO_3_-H]^−^ at m/z 577.13 was detected, which was formed by a neutral loss of 79.95 Da (elemental composition: SO_3_). In addition, the [M-SO_3_-H]^−^ could yield the fragment ions at m/z 407.07 and 425.07, which were the RDA characteristic fragments of procyanidin B2 (shown in Figure 1). As a result, they were identified as procyanidin B2 sulfate. Furthermore, the [M-SO_3_-H]^−^ at m/z 369.02, 216.98, and 137.02 were observed, and sulfate (−)**-**epicatechin preferably occurred at C-5-OH or C-7-OH based on several previous studies. Hence, sulfonic group was concluded to conjugate to the 5/7-OH of A-ring of upper or lower (−)**-**epicatechin unit, however the accurate site of M34, M35, and M36 was unclear (Liang et al., 2013, 2014).

#### Metabolites formed through methylation and glucuronidation: M37

M37 had the molecular formula of C_38_H_38_O_18_, as calculated from their HRMS data. In their NI MS^n^ spectra, the [M-C_6_H_8_O_6_-H]^−^ at m/z 605.16 was observed, which was formed by a neutral loss of 176.03 Da (elemental composition: C_6_H_8_O_6_). Further, the characteristic fragmentation ions at m/z 245.08, m/z 421.10 were noticed, which were formed by the RDA cleavage of (−)**-**epicatechin unit, suggested that the B-ring was substituted by a glucuronic group (Liang et al., 2014). Therefore, the glucuronidation group of M37 was supposed to be in 4′-O-upper (−)**-**epicatechin unit of M32 or 3′/4′-O-lower (−)**-**epicatechin unit of M32.

#### Metabolites formed through methylation and sulfation: M38–M41

In the MS^n^ spectra of M38, M39, and M40, the same molecular ion [M-H]^−^ at m/z 689.12 was found, which yielded the fragmentation ions at m/z 591.15, 407.07, and 289.06. Thus, they were identified to be the metabolites of procyanidin B2. Moreover, the molecular weight were 97.97 Da (elemental composition: H_2_O and SO_3_) larger than the [M-H]^−^ at m/z 591.15 of M31, and the characteristic fragment ions at m/z 451.09, 465.11 were detected in the NI MS^2^ spectra. Hence, the methyl group was lined to the C-3′/4′-OH of lower (−)**-**epicatechin unit, but the exact site could not be determined, and the binding sites of hydration and sulfation were also unclear. In the MS^n^ spectra of M41, the [M-H]^−^ at m/z 703.13 was observed, which yielded the fragment ions at m/z 605.16 and m/z 289.06. Additionally, the molecular weight were 97.97 Da (elemental composition: H_2_O and SO_3_) larger than the [M-H]^−^ at m/z 605.16 of M32. As results, it was presumably identified to be the metabolites of procyanidin B2 of hydration sulfate M32.

#### Metabolites formed through hydroxylation and sulfation: M42–M43

The M42 and M43 showed the same molecular ion at m/z 673.08, which were 16.01 Da (elemental composition: O) larger than molecular ion at m/z 657.09 of M34, suggesting that they contained one additional oxygen atom. M42 and M43 produced an associated RDA fragment at m/z 423.07, 407.07, and a MS^2^ fragmentation ion at m/z 289.06 possibly upon cleavage of the interflavan bond and the release of an unchanged (−)**-**epicatechin unit (Stoupi et al., 2010a). This observation indicated that the oxygen was not inserted in the lower flavanol unit. M42 and M43 were assigned as tentatively as either 8-hydroxy M34 or 6-hydroxy upper (−)**-**epicatechin unit of M34.

#### Metabolites formed through hydration: M44

The molecular formula of M44 were calculated to be C_30_H_30_O_14_ with the [M-H]^−^ at m/z 613.15 based on the HRMS data. The molecular weight of M44 was 36.02 Da (elemental composition: H_4_O_2_) larger than 577.13, which yielded the same characteristic ions as those of the reference standard procyanidin B2. Therefore, M44 was tentatively identified as double hydration metabolite of procyanidin B2.

### Metabolites formed by C-ring cleavage in upper(−)-epicatechin unit of procyanidin B2: M45

M45 had the molecular formula C_31_H_30_O_12_ with the [M-CH_2_-H]^−^ at m/z 579.15 and m/z 289.07 in the NI MS^n^ spectra, which was confirmed as methyl C-ring cleavage of procyanidin B2 based on previous reports. Although there should be two potential sites for reductive cleavage of the C-ring (upper unit and lower unit), the site of upper (−)**-**epicatechin unit was more accessible to reaction (Stoupi et al., 2010b). As results, M45 could be defined as upper C-ring cleavage of M29.

### Unidentified metabolites of procyanidin B2: M46–M53

In NI MS^n^ spectra of all compounds, the [M-H]^−^ at m/z 702.1426, 676.1156 691.1194, 685.1241, 705.1387, 627.1335, 714.1452, and 325.0501 were detected, respectively, which were identified as the metabolites of procyanidin B2 in comparison with the fragmentation behaviors (shown in Table 2). In addition, m/z 591.14 was observed in MS^n^ spectra of M50 and M51, respectively. Hence, M50 and M51 should be the methyl procyanidin B2, but the structure of all compounds consisting of complicated aglucon and misty conjugated sites were not fully identified with pure high-resolution mass spectrometry data in the absence of applicable standard and guidance from previous literature.

### Possible metabolic pathway of procyanidin B2 in mice

The MS^n^ data with high mass accuracy provided much information to tentatively analyze the structure of metabolites under negative ion mode. The primary objective was to characterize the relatively comprehensive metabolites of the procyanidin B2 in mice in the present study. Therefore, we analyzed and compared the metabolites in urine, plasma, and organ tissue samples following oral routes of administration. The base peak chromatograms of the mice urine and plasma samples detected in negative ion mode are shown in Figures 3 and 4, respectively. 53 metabolites of procyanidin B2 (24 new metabolites) showed that the procyanidin B2 was metabolized diversely (shown in Tables 1, 2). Although the previous study reported that glucuronide or sulfate metabolites of dimeric procyanidins had not been detected in biological fluids, we identified the intact procyanidin B2 conjugates of 10 sulfate metabolites and 1 glucuronide metabolites in this work (Appeldoorn et al., 2009a,b). Meanwhile, aromatic acid metabolites were very common in the metabolic pathways of procyanidins after cleavage of the interflavan bond.

Furthermore, a possible metabolic pathway was proposed in Figure 5. The metabolites of procyanidin B2 could be categorized into two major groups: (a) cleavage of interflavanic bond (C4–C8) of procyanidin B2 (M_r_ < 577) and formation of small molecule material, including (−)-epicatechin, C-ring cleavage of epicatechin metabolites, phenylvalerolactone metabolites, and aromatic acid metabolites; (b) metabolites with intact interflavanic bond C4–C8, including procyanidin B2 conjugates, partial heterocyclic ring cleavage products, and their conjugates.

In general, the conjugation process (glucuronidation, sulfation, and methylation) affected the bioactive properties of the parent compound. Sulfation and glucuronidation had been reported to improve a considerable attenuation of biological activity. Additionally, the case of methylation seemed to be more complex because the incorporation of methyl groups reduced the number of available OH group, but increased the lipophilic nature of the compound, which could be advantageous for cellular uptake by passive diffusion (Donovan et al., 2007; Monagas et al., 2010). In particular, we found 32 sulfation metabolites, 3 glucuronidation metabolites, and 14 methylation metabolites, respectively. Besides, M4 and M5 exhibited the potential inhibition of lipid oxidation and inhibition of apoptosis induced by oxidized LDL. M1 was identified as beneficial effects in vascular disaster (Schroeter et al., 2001; Su et al., 2004). In the case of M10, M11, M21, and M22, they have been reported to be a slight increase in antioxidant activity (Grimm et al., 2004). In addition, the biological activities of metabolites of procyanidin B2 were still largely unknown, thus the bioactivities of metabolites were also worthy to further research.

### Distribution of the procyanidin B2 in mice

In this work, the metabolic profile of procyanidin B2 in mice urine, plasma and organs were qualitatively described by UPLC-DAD-ESI-IT-TOF method. The numbers of all detected metabolites in plasma, urine, small intestine, liver, heart, kidney, lung, brain and spleen were 37, 34, 23, 13, 12, 7, 3, 1, and 3, respectively.

According to previous reports, liver had more importance in the metabolization process of polyphenols showing a higher metabolite concentration (Appeldoorn et al., 2009a). In this work, all metabolites found in the liver was M_r_ > 577, which indicated that cleavage of interflavanic bond could not occur in the liver, and conjugation reaction (glucuronidated, sulfated, and methylated) was the major reaction formation with intact procyanidin B2. 34 metabolites in urine and 5 in the kidney showed that the urinary path was the main procyanidin B2 excretion pathway, which was consistent with the conclusion of previous literature (Serra et al., 2011). There were 12 metabolites observed in the heart, which would enhance their described beneficial effects. The presence of products in such tissues as the heart may be related to the potential health benefits of procyanidin B2, especially in the context of cardiovascular health effect (Fraga et al., 2011). 23 metabolites were detected in small intestine, including M1, M4–M5, M10–M11, M16, M19, M22–M23, M29–M32, M34–M36, M44–M45, M46–M49, and M53. Furthermore, 14 metabolites were the conjugation metabolites with intact procyanidin B2, suggesting that the procyanidin B2 could pass intact through the gastrointestinal tract, reaching the small intestine where it was transformed by the intestinal microbiota before absorption, and these metabolites could reach the systemic circulation to be transported to other tissue where they could be further biotransformed according to the previous reports (Aura, 2008; Crozier et al., 2009; Monagas et al., 2010; Margalef et al., 2015).

## Conclusions

It was the first time that the absorption and metabolism of oral administration of pure procyanidin B2 in mice was explored using UPLC-DAD-ESI-IT-TOF in the present study. In total, 45 metabolites of procyanidin B2 were preliminarily identified, and 24 of them were firstly reported. The results showed that the small intestine represented the largest proportion of metabolites in comparison with other tissues, and liver was the major conjugated reaction organ tissue of procyanidin B2. Meanwhile, aromatic acid metabolites were very common in the metabolic pathways of procyanidin B2 after cleavage of the interflavan bond. In conclusion, we profiled the metabolites of procyanidin B2 in mice and revealed the distribution of metabolites. Our findings improve understanding of the metabolism and bioactive action mechanism of procyanidin B2. As for 24 new metabolites, the conjugation types and structure skeletons could be preliminarily determined using the IT-TOF technique. To ascertain their exact structures, the characterization of metabolites of procyanidin B2 *in vivo* should be further confirmed in the future work.

## Author contributions

YX designed the experiments and wrote the paper. ZH and ZY performed the experiments and analyzed data. YZ analyzed data and contributed analysis tools. TL performed the experiments. XZ conceived the experiments and revised the paper. DC analyzed data and revised the paper.

### Conflict of interest statement

The authors declare that the research was conducted in the absence of any commercial or financial relationships that could be construed as a potential conflict of interest.
